# Economic competencies as part of 21st century skills and their relevance to predicting the choice of a study program

**DOI:** 10.1177/14788047251314814

**Published:** 2025-03-13

**Authors:** Michael Jüttler, Stephan Schumann, Nicolas Hübner, Benjamin Nagengast

**Affiliations:** Zurich University of Applied Sciences, Switzerland; 26567 University of Konstanz, Germany; 581937 University of Tübingen, Germany; 233569 University of Tübingen, Germany; Korea University, Korea

**Keywords:** economics education, twenty-first-century skills, study choice, upper secondary education, higher education

## Abstract

The debate surrounding skills that young people will need in the future to master current national and global challenges is subject to a continuous, controversial process. A consensus has resulted in what are known as twenty-first-century skills. These skills aim to enable learners to form well-founded opinions and be educated to become responsible citizens. In this context, significant weight can be attributed to economic competencies, but it remains unclear what role economic competencies play in predicting study program choices. This paper provides a comprehensive discussion of economic competencies as a part of twenty-first-century skills and as a predictor for program choices. To achieve this, we analysed the economic competencies of 1397 Swiss students in different study programs. Based on multinomial regression models, our findings show that students in economics disciplines score higher in terms of economic knowledge and skills. However, considering motivational-affective dimensions of economic competencies, students in several other study programs have similar scores to those of economics students. This paper makes an important contribution regarding economic competencies as part of twenty-first-century skills and their domain-specificity when transitioning from secondary education to university.

## Introduction

The basic skills and knowledge people need to participate in society as responsible and informed citizens is probably one of the most fundamental questions of general education (see, e.g., Zilic, 2018). This question cannot be answered in isolation from the current national and global circumstances and challenges that significantly influence coexistence in a society. Increasing digitalization, artificial intelligence, economic interdependence, globalization, information diversity, heterogeneity, and growing individuality are just a few characteristics significantly shaping our society today (e.g., González-Pérez and Ramírez-Montoya, 2022). Against the background of these demands, skills such as creativity, innovative ability, critical thinking, problem-solving ability, information literacy, communication skills, and ethically responsible thinking and acting, rather than purely specialized knowledge or competence – as has been the case in the past – are becoming the focus of attention (ibid.). Accordingly, these skills are often known as twenty-first-century skills. Many of the challenges mentioned above are not new; indeed, they were already characterized in the perspective scheme within the context of the rationale of educational content by Klafki (1997) as an attempt to solve these epochal problems, still recognized today by numerous other researchers in general education (for an overview, see González-Perez and Ramírez-Montoya, 2022).

It is, therefore, not without reason that there is a fundamental demand in most countries that general upper secondary education must equip students with these necessary skills (e.g., Jansen and van der Meer, 2012; Torenbeek et al., 2010) to qualify them to enter higher education, certifying their general ability to study (e.g., Organisation for Economic Cooperation and Development [OECD], 2019). Thus, the discussion about twenty-first-century skills is essential not only at the level of general upper-secondary education but also at the level of tertiary education. This is especially important against the background that students with a general ability to study not only gain a high level of general education in the future but often possess a leading position with high (societal) responsibilities (e.g., human resource management, economic decision-making power, etc.).

Against this outlined background, many researchers suggest that basic economic education is a crucial component of this general education at both upper-secondary and tertiary levels (e.g., Brückner et al., 2015; Aprea et al., 2016; Schumann and Eberle, 2014; Siegfried, 2019; Walstad, 1998; Wuttke et al., 2016). As a result, the relevance of economic education for society and industry has increased consistently in recent decades (e.g., Aprea et al., 2016; Siegfried, 2019; Walstad and Watts, 2015). Economic education has now become a central feature of general school education in different countries (e.g., Germany and Switzerland). In this context, empirical research conducted thus far has identified a substantial lack of both financial and economic competencies among students in many countries worldwide, particularly among upper-secondary students (Aprea et al., 2016; Council on Economic Education [CEE], 2014; Lusardi and Mitchell, 2014; OECD, 2014; Scheer, 1974; Walstad, 1994, 1998; Walstad and Rebeck, 2001). Most researchers exclusively cite financial knowledge (or financial literacy) as an essential part of twenty-first-century skills at both individual and societal levels (see, e.g., Erol, 2021; Hussain and Sajjad, 2016; Lusardi, 2015; Lusardi and Messy, 2023). To the best of our knowledge, the specific role of economic competencies (other than financial literacy) in twenty-first-century skills has never been examined in detail.

The first objective of this study is to explain how economic competencies can be understood as part of twenty-first-century skills (beyond financial literacy) and how students of different fields of study differ regarding the economic competencies they possess at the end of secondary education. Considering previous research on this first objective, most studies to date have been limited to examining basal skills, such as language and mathematics achievement, and focus only on one level of education. This suggests an explicit limitation and does not represent the competencies necessary for responsible participation in society (for a list of the essential competencies, see the explanation of twenty-first-century skills in the theoretical section) and the relevance of these competencies for subsequent educational decisions. In addition, the extent to which prospective students gain these competencies through their general school education and to which university programs they lead is still unclear.

In addition to economic competencies being necessary as part of general school education at the upper-secondary and tertiary levels, they must also be attributed a domain-specific role in the transition from school to university and thereby in the choice of subject at the university level. The lack of economic competencies at the secondary level mentioned above is significant because economics^1^ is one of the most popular fields of study at universities worldwide (e.g., German Federal Statistical Office [German FSO], 2017; Higher Education Statistics Agency, 2018; National Center for Education Statistics, 2016; Swiss Coordination Centre for Research in Education, 2023). Given this fact, surprisingly little is known about the transition to an economics study program (particularly in comparison to research in the field of STEM; see, e.g., Parker et al., 2012; Wang, 2013; Wang and Degol, 2013; Wang and Degol, 2017). Research in the field of STEM shows that (domain-specific) achievement and interests, as well as course choices in upper secondary school, are some of the main predictors for choosing one of these subjects at university.

Thus, the second objective of this paper is to answer the question of how economic competencies influence the choice of a study program after leaving upper secondary school.

The combination of the first and the second study objectives offers a platform for discussing and comparing economic competencies as both a twenty-first-century skill and a domain-specific predictor of choosing a particular study program. The overarching question, therefore, is to what extent economic competencies based on socially relevant economic knowledge and skills act exclusively as a predictor for the choice of a degree program in economics or whether and to what extent economic competencies can also be found in other groups in terms of relevant twenty-first-century skills. To achieve this, our paper will present a comprehensive conceptual understanding of economic competencies based on current economic and societal problems rather than curricular, subject-related definitions of educational institutions, the professional requirements of a job, or a limited focus on private financial decisions (e.g., investing or saving money). Based on this conceptual understanding, economic competencies will be theoretically justified as part of twenty-first-century skills. For this paper's second objective, the expectancy-value model by Eccles et al. (1983) and Eccles and Wigfield (2002, 2020) will be described, and economic competencies will be implemented into this model. Since a sample of Swiss students is used, our paper focuses on transitions from school to university in this country. Based on the presented objectives of the study, specific research questions will be derived. Next, the methodological background of the study is comprehensively described before reporting the empirical results for (a) students’ economic competencies and (b) the predictive power of economic competencies in terms of the choice of study program. Finally, the results will be discussed, and the research questions answered. On this basis, the analyses of economic competencies for different student groups provide an essential basis for considering specific factors for the promotion of economic competencies and evaluating the importance of economic competencies as part of twenty-first-century skills.

## Theoretical Background

### The meaning of twenty-first-century skills from an educational perspective

The competencies and skills a society requires to cope with current and future national and global challenges are in constant discourse (e.g., González-Salamanca et al., 2020; Chalkiadaki, 2018). Several unique challenges are emerging for the twenty-first century. These include, among others, an expanding economy and globalization, growing economic and cultural uniformity, threats to our ecosystem (e.g., climate change), increasing individualization and social diversity, the rapid dissemination of and unlimited access to knowledge and information, and the increasing spread of artificial intelligence and related fast technological development (see, e.g., Germaine et al., 2016; Ghaith, 2010; González-Salamanca et al., 2020). These challenges strongly impact international relations between countries, the individuals in society and their coexistence, the labor market, and future demands on the workforce (e.g., Germaine et al., 2016). This altered industrial working environment is often described as Industry 4.0 (see González-Pérez and Ramírez-Montoya, 2022). Consequently, researchers ask what competencies and skills people will need as citizens and workers in the future and what competencies a society needs from its citizens to be viable (the so-called twenty-first-century skills). From an educational perspective, the question arises as to which curricular (or content-specific), didactic-methodical, and educational adaptations and modernizations are necessary to teach these skills comprehensively – often referred to as Education 4.0 (ibid.). Numerous research groups have worked on identifying twenty-first-century skills, and a consistent picture emerges in which various categories of these skills are grouped (see, e.g., the definitions by the National Education Association, 2012, or the Partnership for twenty-first Century Skills (P21), 2011). These include (1) critical thinking and problem-solving, (2) communication skills, (3) collaborative skills, and (4) creativity and innovation skills. This categorization is very general, and numerous works exist that further differentiate these (see, e.g., Germaine et al., 2016).

Furthermore, previous definitions have been criticized for various reasons, as they have been too strongly influenced by economy and industry, take too little account of emotional-affective factors, are too broad and unclear, and completely disregard aspects relevant to the learning process (such as the enjoyment of learning) (e.g., Daumüller and Wisniewski, 2023; for an overview see also Germaine et al., 2016). Finally, there is the question of whether the skills mentioned here are genuinely new at all. Silva (2009: 631) said they were not but suggested they are “newly important.” This is justified by the fact that making well-informed and critically reflected decisions is becoming increasingly important in light of the challenges mentioned above, such as the unlimited access to information and the high demand for responsible behavior. Larson and Miller (2011) trace this basic idea back to the considerations of John Dewey, who believed education should be experience-based and closely interact with the real environment. Accordingly, it is an essential task of each domain to examine the extent to which they can contribute to the promotion of twenty-first-century skills and be integrated into the future experiences of young people. Such an integration is explained below for the economic competencies that are the focus of this paper.

### Underlying definition of economic competencies as socially relevant basic competence

Before defining economic competencies^2^, it is necessary to understand that various conceptual definitions must be differentiated. Wuttke et al. (2019) identify at least four concepts: (1) economic competencies as economic literacy (civic-economic competence), (2) economic competencies as financial literacy, (3) economic competencies as general business-related competence, and (4) economic competencies as financial accounting-related competence. Concepts (3) and (4) can also be combined as vocational-commercial competence.

Our paper focuses on the first concept of economic competencies. This has a relatively long tradition and concentrates on a general economic education, which all people in modern society require to function as responsible citizens and make well-founded decisions about topical societal issues (e.g., Beck, 1989; Soper, 1978, 1979), such as unemployment, social security, business ethics, and corporate social responsibility. Accordingly, this is closest to the concept of an economic education. A limitation of previous definitions of this concept is that these are generally reduced to basic economic knowledge and exclude business administration and financial accounting, as well as the affective-emotional, motivational, and value-oriented dimensions of economics (e.g., Beck, 1989; Wuttke et al., 2019; for a comprehensive discussion see Schumann and Eberle, 2014). Previous research on economic literacy points to a clear knowledge deficit among students at both secondary and tertiary levels (e.g., Rudeloff, 2019, Walstad and Rebeck, 2001).

The first concept must be separated from the second (financial literacy), which focuses primarily on (private) financial decisions such as dealing with personal assets, private insurance, loans, investments, and retirement planning (e.g., Retzmann and Seeber, 2016). A more comprehensive understanding is supported by the OECD (2014), including societal topics (Retzmann and Seeber, 2016). These topics are often also included in definitions of economic literacy, which leads to the conclusion that financial literacy is frequently understood as a critical part of economic literacy (Wuttke et al., 2019). Similar to economic literacy, many studies point to a severe deficit of financial understanding among adolescents and adults (e.g., Aprea et al., 2016; Lusardi and Mitchell, 2014; OECD, 2014).

These two concepts can further be distinguished from the third and fourth concepts, which focus on commercial competencies (e.g., Seeber et al., 2015; Winther, 2010). While economic competencies follow a typical literacy concept focusing on general economic education, commercial competencies focus on mastering vocational issues (see Seeber et al., 2015). Thus, both concepts have different relations in terms of their domain. While economic competencies are more subject-oriented, commercial competencies are more task-oriented and have a vocational context (ibid.).

In this study, we focus on a multidimensional understanding of economic competencies, and the following facets are included:

1. **Economic knowledge and skills** required to solve economic issues and to evaluate suggested solutions to economic problems.
2. **Motivation** to address and **interest** in economic issues.
3. **Attitude toward economics** and **value-oriented dispositions** to solve economic issues reflectively and evaluate solutions appropriately.

In this definition, we follow the conceptualization of Weinert (2001). As mentioned, this definition corresponds to economic literacy (e.g., Scheer, 1974; Soper, 1978, 1979; Soper and Walstad, 1987; Wuttke et al., 2016). Against this background, economic competencies are seen as necessary to allow individuals to shape their own lives and foster their social participation in modern societies (Schumann et al., 2017). They are not explicitly conceptualized as curricular tests, tests for professional ability, or tests of the ability to study economics (e.g., Graduate Record Exam subject tests), which are used to predict academic performance (Kuncel et al., 2001). Instead, the emphasis is on typical economic issues in modern societies (e.g., understanding the causes and consequences of national debt or the relationship between fixed and unit costs). The different facets of the economic competencies mentioned above are explained in detail below.

1. Economic knowledge and skills form the core dimension of economic competencies. While most definitions of economic knowledge only consider economics in a narrow sense (see, e.g., Test on Economic Literacy (TEL), Happ and Zlatkin-Troitschanskaia, 2021), we also include business administration and accounting as further dimensions (see, e.g., Schumann and Eberle, 2014). Additionally, this definition includes characteristics beyond the domain-specific cognitive performance dispositions (motivational-affective dispositions; for details, see ibid.). These important professional skills and problem-solving abilities predict academic outcomes (e.g., Gutman and Schoon, 2013; Rosen et al., 2010) and will be explained in the following.
2. Motivation focuses on how students are intrinsically motivated to solve economic issues and is consistent with self-determination theory (Ryan and Deci, 2000). Against this background, intrinsic motivation is process oriented. In contrast, interest is object-oriented and consistent with person-object theory (Krapp, 1993). Interest and intrinsic motivation are strongly related (ibid.): Someone who is interested in a topic or subject is more likely intrinsically motivated while solving a specific task and vice versa. Although they are dependent, a distinction in different contexts is required. Since interest is object-related, it can be used, e.g., to explain the choice of a study program (see Eccles and Wigfield, 2002). In contrast, through its process orientation, intrinsic motivation can be used to infer, e.g., reasons for engagement and persistence in an activity (see Ryan and Deci, 2000). Thus, both represent important theoretically separate parts of economic competencies.
3. Attitudes toward economics describe students’ willingness to tackle economic issues and their self-confidence in solving such problems (Beck, 1989; Walstad and Soper, 1983). This also comprises students’ perceptions regarding the general importance of economics. Value-oriented dispositions address students’ perceptions of being able to reflect morally on solutions to economic issues and act responsibly. Value-oriented dispositions are a vital dimension of economic competence because by neglecting it, economically competent persons would also include white-collar criminals (see Beck, 1989).

### Economic competencies as part of twenty-first-century skills

Developing economic competencies while still at school is a crucial aspect of general education and is indispensable to the development of societies in the twenty-first century for several reasons (e.g., Aprea et al., 2016; Beck and Krumm, 1994; Walstad, 1994; Walstad and Watts, 2015):

First, from an individual's perspective, economic education goes far beyond understanding simple economic principles, such as the economic cycle or the function of supply and demand. To address future economic challenges, students must learn to make sound decisions, acting as informed citizens within complex economic environments (Schumann et al., 2017; Walstad, 1994; Wuttke et al., 2016). Second, there is a growing number of studies showing the positive effects of economic (or financial) education on personal (financial) well-being (e.g., Kaiser and Menkoff, 2017; Lusardi et al., 2017; Walstad et al., 2010). Third, strong empirical evidence exists of the predictive power of school-related competencies for long-term learning outcomes (e.g., academic success) within a domain (e.g., Robbins et al., 2004; van der Zanden et al., 2018). Fourth, against the background of these positive long-term effects and the growing number of students in economics, an important question is whether the more vigorous development of economic competencies through school positively affects students’ decisions to study economics and their later academic success in economics. Many studies point to the positive effects of earlier exposure to economic education (Brückner et al., 2015; Happ et al., 2018; Walstad et al., 2010; Walstad and Watts, 2015; Yamaoka et al., 2010). Finally, a wide range of occupations require economic competencies (Varga et al., 2016), so economic education in secondary school prepares students for university as well as the labor market.

Accordingly, as Siegfried (2019) pointed out, economics is integral to general education. In this context, she emphasizes knowledge of, attitudes toward, and value-oriented dispositions regarding economics as essential factors guiding and regulating behavior, which are also considered central to responsible economic participation in other study programs (e.g., Allgood and Walstad 1999).

In addition to the individual level, previous research also assumes the positive effects of economic competencies on a societal level (Aprea et al., 2016; Walstad and Rebeck, 2002; Wuttke et al., 2016). A central task of educational institutions is to provide students with a basic understanding of societally relevant topics to help them become socially responsible and critically reflective citizens. As mentioned before, many reports in international studies show a considerable lack of economic understanding among secondary school students (e.g., see Happ and Zlatkin-Troitschanskaia, 2021; Walstad, 2001; Walstad and Rebeck, 2008). In an international comparison, Western countries perform significantly below average compared to Asian countries (for an overview, see Jüttler and Schumann, 2022a). Based on these deficits in foundational economic education (e.g., Wuttke et al., 2016), whether school leavers can understand socially relevant, key economic issues must be questioned. Compensating for this is essential to enabling comprehensive social opinion formation and participation (ibid.).

In summary, in light of twenty-first-century skills, economic competencies must be considered vital, especially regarding critical thinking and problem-solving skills. Against this background, researchers strongly suggest the need for general economic understanding, a positive attitude toward economics, and a robust and value-oriented disposition regarding economics to form considered opinions about economic issues (see also Siegfried, 2019; Wuttke, 2008).

However, in addition to the role of economic competencies as part of twenty-first-century skills, economic competencies must also be seen as domain-specific competencies that affect domain-specific subject choices. This matter is addressed in the second part of this paper and is explained in the following section.

### Study choice, school education, and the role of domain-specific competencies

Study choices are complex, momentous educational decisions that depend on many individual and contextual variables (for an overview, see, e.g., Quadlin, 2020). A well-established theory in educational psychology is expectancy-value theory (EVT; Eccles et al., 1983; Eccles and Wigfield, 2002, 2020). There is strong empirical evidence of the effects of (school) achievement and interests on educational decisions (for reviews, see Hidi and Renninger, 2006; Krapp, 2002; Schiefele, 2009; Wang and Degol, 2013; Wigfield and Cambria, 2010a, 2010b). Students who perform better, have higher expectations of success and are more interested in a specific domain are more likely to choose a corresponding subject (Eccles and Wigfield, 2002). In addition, (school) achievement and interests are related (e.g., Eccles and Wigfield, 2002; Marsh et al., 2005) and converge over (school) time (e.g., Reeve and Hakel, 2000; Wigfield and Cambria, 2010b). Eccles and Wigfield (2002) pointed out how crucial school education and school experiences are regarding study choices and successful completion. Specifically, upper secondary-level schools significantly influence a student's general ability to study, including domain-specific skills, abilities, interests, and academic self-efficacy – all relevant predictors for study choices (ibid.). Mismatches between the requirements of a particular study course and student competencies often lead to lower academic performance and higher dissatisfaction, followed by dropouts (e.g., Richardson et al., 2012; Robbins et al., 2004; Tinto, 1993). Against this background, domain-specific competencies after school can be seen as a significant predictor of postsecondary subject choices (for an overview of STEM, see, e.g., Wang and Degol, 2013). Since previous research has focused almost exclusively on STEM, examining other less-frequently considered areas of social studies (such as economics or political science) is even more critical. This is also important to prove the generalizability of these theoretical considerations, especially because the development of the EVT model has already been made concerning mathematics education.

In addition to (domain-specific) competencies, further background variables are key predictors of subject choices. First, there is a large body of research on gender effects on subject choices, indicating a strong influence of gender stereotypes (Eccles and Wigfield, 2002; Wang and Degol, 2017). Second, the social status of the family is another significant predictor of study choices (e.g., status maintenance; Leppel et al., 2001; Parker et al., 2016), meaning that students tend to choose a study program that leads to an occupation at least as advanced as that of their parents (Stocké, 2007). Finally, students’ earlier schooling plays a crucial role (Eccles and Wigfield, 2002), showing that experiences in school (e.g., grades or advanced courses selected) directly or indirectly influence their subsequent subject decisions.

### Effects of economic competencies on study choices

In the context of the transition from school to university, almost no empirical research exists on economics. The few existing studies in this field found financial rewards, job availability, and interest in the subject and career to be the most important predictors of study choices (for a literature review, see Simons et al., 2004). However, most of these studies used cross-sectional designs and did not implement competence tests of students at the end of compulsory schooling. A growing body of studies on the effects of economic competencies on study choices has mainly focused on the importance of earlier economic education on academic achievement (Brückner et al., 2015; Happ et al., 2018; Zlatkin-Troitschanskaia et al., 2013). However, these studies only considered individual characteristics at the end of upper secondary education (e.g., their economic knowledge and skills) to a limited extent.

Although some empirical studies focus on U.S. students, most relate to financial literacy and its impact on college attendance, finding that students with higher financial literacy are generally more likely to attend college (see e.g., Mandell, 2008). Additionally, studies focusing on business students have found subject-specific interest to be the main reason for choosing this study program (e.g., Malgwi et al., 2005). These studies also used cross-sectional designs and retrospectively asked university students why they chose the study program. Furthermore, they used questionnaires instead of more extensive and reliable achievement tests to assess aptitude (or skills).

To date, it is still unclear exactly how economic competencies contribute to explaining the transition from school to university. Specifically, it is unclear whether students with higher economic competencies actually end up in economics study programs, unlike other predictors, such as verbal and mathematics skills^3^. Indeed, studies that observe the presence of economic competencies among different groups of students in the context of twenty-first-century skills are, to our knowledge, rare or nonexistent.

## Transitions from School to University in Switzerland

When focusing on educational transitions, it is important to explain the specific characteristics of the country in question; our study relies on data collected in Switzerland. In Switzerland, at least two pathways lead to a university or a university of applied sciences – a general educational and a vocational pathway (see also SCCRE, 2023). The general educational pathway through grammar schools (in Switzerland: Maturitätsschule, GS) represents the traditional major pathway from school to university, whereby GS students meet a general qualification to enter university. The vocational path is relatively “new” and was formed in the 1990s by the establishment of universities of applied sciences that are more vocationally (or practically) oriented and require practical experience of at least one year in a given study program (e.g., business administration; for detailed information see, e.g., Gonon, 1994, 1997, 2013). Students following the vocational track can complete a federal vocational baccalaureate (FVB) simultaneously or after their apprenticeship by attending an FVB school (for more information on these types of hybrid qualifications, see Deissinger et al., 2013). Since this study only focuses on general education and only about two percent of an FVB cohort progresses to a university, this track will be excluded from our analysis.

Over 90 percent of a GS cohort goes on to higher education (SCCRE, 2023). GS students must choose, in addition to other subjects, economics and law as an advanced course (four to six lessons per week, depending on the administrative region) or as an introductory course (one to two lessons per week), enabling them to gain at least a basic education in this domain^4^.

There is also a third pathway that leads to higher education via what are known as upper-secondary specialized schools. Like an FVB, these comprise only a relatively small percentage of university admissions and are often domain-related (ibid.). Thus, this route to university is also excluded from our study.

## Research Question

Based on the theoretical considerations, we are posing two research questions that correspond to the two research objectives formulated in the introduction:

1. How do the economic competencies as part of twenty-first-century skills differ between students in different study programs?
2. How do students’ economic competencies at the end of upper secondary school predict their choice of an economics study program?

The first research question discusses economic competencies as essential to students’ twenty-first-century skills. To date, no data exists showing which programs students with different economic competencies choose to take. This question is vital because it shows that economic competencies cannot be considered solely domain-specific competencies (see second research question below). This would underline previous assumptions about the importance of economic competencies as part of general education. Furthermore, the analyses provide a crucial basis for identifying and discussing the availability of different degrees of economic competencies – especially in the area of knowledge and attitude – specifically according to various study groups. If there are substantial differences between non-economics programs, the educational question can at least be raised as to whether interdisciplinary courses in economics should be offered to a greater extent in specific higher education programs. Considerations regarding greater interdisciplinarity would also be conceivable. Since it is only in recent years that economic problems have increasingly been regarded as occupational and general societal problems, there is still a shortage of evidence – in contrast to mathematical and verbal skills – on the development of economic competencies at different levels of education.

In addition to discussing economic competencies as part of twenty-first-century skills and economics education as part of general school education, the second research question focuses on economic competencies as a domain-specific competence that predicts the choice of an economics study program. To answer this research question, we follow EVT. Considering that subject courses are strongly determined by self-selection processes and that there are no systematic constraints within the Swiss education system to choose a study subject (see SCCRE 2023), it is expected that students with higher economic knowledge and skills and higher values of further facets (interest, intrinsic motivation, attitude and value-oriented dispositions) regarding economics are more likely to choose to study economics than other fields of study, controlling for other relevant variables. In contrast, it is assumed that students with higher mathematical or verbal achievements are more likely to choose different fields of study, such as natural sciences or humanities, controlling for other relevant variables. This is because mathematical achievement is more immediately needed in exact, natural, and technical sciences (ENT), while verbal achievement is more immediately sought after in humanities or social sciences (see, e.g., Wang and Degol, 2013). This leads to the following hypotheses:

(H1) Students with higher economic competencies at the end of their upper secondary education are more likely to choose an economics study program than another study program.

(H2) Students with higher mathematical achievement are more likely to choose exact, natural, or technical sciences than economics.

(H3) Students with higher verbal achievement are more likely to choose humanities or social sciences than economics.

In addition to economic competencies, it is assumed that students who chose the advanced “Economics and Law” course at school (for more information on advanced courses, see also the description of the Swiss Education System in the previous section) had a greater intention to study economics and were thus more likely to choose economics at university, regardless of their economic competencies. This leads to the fourth hypothesis:

(H4) Students who took the advanced “Economics and Law” course at secondary school are more likely to choose to study economics than students who took another advanced course.

## Methodology

### Design

As mentioned before, this study was conducted in the German-speaking part of Switzerland. The analyses are based on a study with two measurement points (T1 and T2). T1 was in the spring/summer of 2011, while T2 was five years later in the spring/summer of 2016. At T1, students’ economic knowledge and skills in their last year of GS were assessed shortly before they graduated. In addition to economic competencies, academic abilities (mathematics, first language, and cognitive ability) were tested, and further facets of economic competencies (interest, intrinsic motivation, attitude, and value-oriented disposition), as well as contextual variables, for example, advanced school course, were assessed via a questionnaire. At T2, information on educational decisions (especially study choices) was collected for the previous five years using a 45-min online questionnaire and a 10-min computer-assisted telephone interview (CATI). CATIs were conducted by trained interviewers at the survey lab of the University of Konstanz^5^. The CATI was used to verify the information gained by the online questionnaire, especially information on educational decisions, including study choices. This was done to ensure the accuracy of data about educational decisions.

### Sample

#### Sampling

The target population included all GS students in the German-speaking part of Switzerland who were about to obtain their leaving certificate in the summer of 2011; this target population included 584 GS classes (Angelone and Berger, 2011). This population was subdivided by advanced courses (explicit strata): 1) GS students with an advanced course in economics and law (GS Economics and Law) and 2) GS students with another advanced course (GS Other). In addition, implicit strata were built according to gender, canton, and class size (ibid.). Based on this, 50 GS classes for each explicit stratum (1838 students) were randomly selected.

In total, *N* = 1277 students from 79 classes participated. Considering the different strata mentioned above, classes that did not participate in the study did not differ systematically from those that participated (ibid.). Because the subsamples for advanced courses were not drawn proportionally to the distribution within the population, stratum-specific weighting was necessary (ibid.). Table A1 in the Appendix provides an overview of the weighted original sample at T1. Students at this point were between 18 and 19 years of age on average.

Of the 1277 students at T1, 947 students agreed to participate in a follow-up study and provided contact information (email and physical addresses). At T2, of the 947 addresses, 728 were still valid. Based on these addresses, 367 GS students participated in T2. Overall, 910 GS students from T1 dropped out. Of the 367 GS students, 320 (approximately 87 percent) were contacted by telephone and participated in CATI.

#### Dropout at T2 and weighting

To examine systematic dropout (unit nonresponse), the samples at T1 and T2 were compared with respect to advanced course, gender and age, as well as individual cognitive variables (e.g., economic knowledge and skills), motivational-affective variables (e.g., interest in economics), and other contextual variables (e.g., social background). There were moderate differences in cognitive variables (e.g., economic competencies, mathematics performance), with students who participated at T2 consistently showing better results than those who did not (see Table A2 in the Appendix).

These selection biases necessitated individual weightings (in addition to the previously calculated stratum-specific weighting). The weighting prevents systematic errors in the results due to the probability of dropout based on the observed variables. Inverse probability weighting was used to calculate these individual weightings (see Brick and Montaquila, 2009; Hong, 2015; Thompson and Arah, 2014). The individual weightings were then used as the reciprocal value of the probability of remaining in the sample. In addition, trimming was used to prevent overweighting (for details, see Kish, 1992). Finally, the individual weightings were combined with the stratum-specific weightings to reproduce the sample's representativeness. Table A2 in the Appendix summarizes the unweighted and weighted means and standard deviations. Comparing the weighted means and standard deviations of students who participated at T2 with those of the unweighted sample of T1, the effect sizes are all small or close to zero, showing that no statistical biases can be assumed based on student participation. Table 1 provides an overview of the weighted longitudinal sample.

**Table 1.** Longitudinal sample (weighted).

	Classes	Students	Gender		Age	
			Female	Male	M	SD
GS (Economics and Law)	36	193	78 (40%)	115 (60%)	23.6	0.7
GS (Other)	41	1204	746 (62%)	458 (38%)	23.5	0.9
Total	77	1397	824 (59%)	573 (41%)	23.5	0.8

*Notes: GS* *=* *grammar school, M* *=* *mean, SD* *=* *standard deviation.*

### Instruments

Table 2 provides an overview of the instruments used at T1.

**Table 2.** Instruments used to measure competencies.

Variable		Items	Reliability	Source
Economic Competencies	Economic Knowledge and Skills	111^a^	0.75^1^	Internal Development (Schumann and Eberle, 2014)
	Interest	3	0.77^2^	Eberle et al., (2009); Prenzel et al. (1996)
	Intrinsic Motivation	4	0.82^2^	Eberle et al. (2009); Prenzel et al. (1996)
	Value-oriented Disposition	9	0.76^2^	Eberle et al. (2009)
	Attitude	14	0.90^2^	Beck (1993)
Mathematic Achievement		59^b^	0.81^1^	Eberle et al. (2008)
First Language Skills		91^b^	0.81^1^	Eberle et al. (2008)
Cognitive Ability		45^b^	0.78^1^	Heller and Perleth (2000)

*^1^WLE reliability, ^2^Cronbach's Alpha;*

*^a^Multi-matrix booklet design with six booklets, ^b^Multi-matrix booklet design with four booklets.*

*Economic competence:* An in-house achievement test was used to measure economic knowledge and skills. The test comprised 111 items that measured three underlying dimensions (economics, business administration, and accounting) in a multi-matrix booklet design with six test booklets. The development of the items is based on a comprehensive media analysis. Approximately 1400 newspaper articles with approximately 30,000 economic terms and concepts were analyzed. These terms could be allocated to the following three areas– business administration, accounting, and economics. The test consisted of multiple-choice and open-ended questions, in which most of the items were multiple-choice questions and just five items were open-ended. To tackle the questions in a given context, students were first introduced to a modified newspaper article. Based on each modified newspaper article, the students answered four to eight items that were partly related to this newspaper article. The six test booklets comprised 21 modified newspaper articles. Examples of closed and open-ended questions are in Appendices A1 and A2.

The three-dimensional solution was not superior to a one-dimensional solution because of high latent correlations between economics, business administration, and accounting (see Schumann and Eberle, 2014). Therefore, the study refers to the one-dimensional solution that shows sufficient reliability (see Table 2). Detailed information about the test construction can be found in Schumann and Eberle (2014), and Schumann et al. (2011).

To measure interest in and intrinsic motivation for solving economic problems, the scales by Prenzel et al. (1996) were used and adapted to the subject of economics and law (Eberle et al., 2009; example items (translated) included the following: *Interest*: “During lessons in economics and law, I often find interesting topics that I want to talk about with others”; *intrinsic motivation*: “During lessons in economics and law, time often flies by”). The measurement of the *value-oriented dispositions* construct originates from Eberle et al. (2009): an example item (translated) is “Lessons in economics and law help me to form my own point of view of economic problems in society.” Items for these three scales were ranked on a four-point Likert scale (1 = does not apply to 4 = applies), whereas the fourth facet – attitudes toward economics – was assessed using a five-point Likert scale (1 = disagree to 5 = agree). A German translation and adaptation of the “Attitudes toward Economics” scale by Walstad and Soper (1983) was used (see Beck, 1993; example item (translated): “Learning economics is a waste of time”).

*Academic and cognitive abilities:* Students completed achievement tests in mathematics (Eberle et al., 2008), first language (ibid.), and cognitive ability (KFT 4-12R by Heller and Perleth, 2000). The total duration for these tests was 90 min. The tests for mathematics and first language ability originated from a nationwide evaluation study in Switzerland (for detailed information on these tests, see Table 2 and Eberle et al., 2008). The three tests were implemented in a multi-matrix booklet design.

*Control variables:* The following control variables were included in the analyses: (1) gender, (2) socioeconomic background (highest international socio-economic index of occupational status (HISEI) of parents; Ganzeboom et al., 1992; International Labor Office [ILO], 2012; Statistik Austria, 2014), (3) advanced courses (economics and law vs. other advanced courses), and (4) school grades at the end of upper secondary education (in economics and law, mathematics, and first language). School grades in Switzerland are coded from 6 = very good to 1 = insufficient, so higher numbers reflect better performance.

*Study choice:* Study choice refers to the first chosen field after leaving school. Depending on the categorization of the Swiss Federal Statistical Office (FSO), the approximately 900 study programs (subjects) were categorized into six groups (Swiss Federal Statistical Office, 2018b, 2019): (1) humanities and social sciences (HS), (2) economics (EC), (3) law, (4) medicine (Med), (5) exact, natural, and technical sciences (ENT) and (6) others. The last group comprises fields of study that are either relatively small or difficult to allocate to larger groups. The most common groups within the “other” category are sports science, teacher training, and specialized fields of study, for example, military sciences. Here, EC is broad, ranging from business administration, accounting, and political economics to interdisciplinary and specialized courses (e.g., information management or tourism).

Since the period between T1 and T2 is five years, we do not know precisely when students chose their first subject field following secondary school. This limitation will be explicitly discussed in the final section of this study.

### Missing values

Missing values (item nonresponse) were mainly found in mathematics, first language, and cognitive ability, and the missing values were approximately 30 percent at individual and class levels. Hence, multiple imputation using chained equations was implemented for the longitudinal sample using a multilevel imputation model with at least seven predictors per outcome variable. Overall, twenty imputed datasets were generated (Rubin, 1987; van Buuren and Groothuis-Oudshoorn, 2011). The imputation model included school class (as a cluster variable) and the calculated individual and stratum-specific weightings. Within the imputation model, both fixed and random effects and class means of cognitive variables were considered. The imputation was performed using the mice.2l.pan function in the R package *mice* (van Buuren and Groothuis-Oudshoorn, 2011).

### Analyses and nested data structure

The test items for economics, mathematics, first language, and cognitive ability were obtained using the one-dimensional Rasch model (Rasch, 1980) with ConQuest software (Wu et al., 2007). Mean-weighted likelihood estimates (WLEs) were used to obtain individual abilities (Warm, 1989). All tests show good reliability (see Table 2).

Individual school grades were standardized by class mean. This adjustment was necessary since class means vary for many reasons (e.g., higher performance within a class, different teachers, or different schools).

To answer the research question, (M)ANOVAs and multinomial logistic regressions were calculated using the weighted and imputed dataset and Mplus software (Version 8; Muthén and Muthén, 2007). Specifically, maximum likelihood estimation with robust standard errors (MLR; sandwich estimator) was used to estimate the model parameters. Regarding multinomial logistic regression, the influence of the predictors within our model (economic competencies, school grades, etc.) on the probability of choosing economics instead of another study program, for example, HS or ENT, was calculated. The class variable was used as a cluster variable to adjust standard errors for the nested data structure. The study program after leaving a GS was used as the nominal dependent variable, with EC as the reference group.

## Results

### Bivariate differences in competencies among study groups

The distribution of the six study groups is shown in Table 3. Compared with the statistical data of the FSO, there were minor differences in the proportions of all university students in Switzerland (Swiss Federal Statistical Office, 2018a), ranging from 0.9 percent (in Med) to 6.8 percent (in ENT). On average, the difference in proportions was 3.2 percent.

**Table 3.** Distribution of the sample (weighted) regarding study choice by subgroup and gender.

Study program	GS (Economics and Law)	GS (Other)	Female	Male	Total
Humanities and social sciences	29	237	195	71	266 (19%)
Economics	72	174	85	161	246 (18%)
Law	21	81	73	30	102 (7%)
Medicine	6	104	60	50	110 (8%)
Exact, natural, or technical sciences	23	210	113	120	23 (17%)
Other	21	287	242	65	308 (22%)
No studies	20	111	55	76	131 (9%)
Total	192	1204	823	573	1396

*Notes: GS* *=* *grammar school.*

Table 4 shows the correlations between (cognitive and noncognitive) individual variables. Except for the relatively high correlations between interest, motivation, attitude, and value-oriented dispositions regarding economics, all other correlations were relatively low or close to zero.

**Table 4.** Correlations between individual (cognitive and noncognitive) variables.

	(1)	(2)	(3)	(4)	(5)	(6)	(7)	(8)	(9)	(10)	(11)	(12)	(13)	(14)
Economic knowledge and skills (1)	1	.17	.11	.30**	.20**	.03	.16**	.23**	.05	.13**	.21**	−.14**	.29**	.26**
Interest in economics (2)		1	.74**	.65**	.55**	.01	−.04	.06	.06	.02	.29*	.09	.06	.20**
Intrinsic motivation in economics (3)			1	.65**	.37**	.05	.00	.07	.05	−.05	.29*	.06	.04	.08
Attitude toward economics (4)				1	.49**	.06	−.06	.09	.08	.01	.32**	.07	.18**	.22**
Value-oriented disposition regarding economics (5)					1	−.02	−.01	.00	−.03	.10	.17*	.07	.13*	.17**
Mathematical achievement (6)						1	.07	.35**	.34**	−.07	.09*	−.05	−.10*	−.08*
First language skills (7)							1	.19**	.06	.26**	.10**	−.06	−.05	−.17**
Cognitive ability (8)								1	.18**	.06	.13*	−.07	−.05	.09*
School grade: Mathematics (9)									1	.07*	.22**	−.01	.01	−.09**
School grade: German (10)										1	.22**	.07	−.10*	−.07**
School grade: Economics and law (11)											1	−.02	−.07*	.05
HISEI (12)												1	.01	−.01
Advanced course (0 = Other, 1 = Economics and Law) (13)													1	.21*
Study choice (0 = Not economics, 1 = Economics) (14)														1

Notes: *p *<* 0.05, **p *<* *0.01; HISEI: highest international socioeconomic index of occupational status (of parents).*

Table 5 shows the means and standard deviations of student competencies, school grades, and socioeconomic background by study program. Since the values are z-standardized, values below 0.0 represent below-average scores, while values over 0.0 represent above-average scores. Considering the five facets of economic competencies, economics students achieved the highest scores (except for intrinsic motivation). Considering mathematical performance and cognitive abilities, economics students were below average compared to students in other fields. For school grades, economics students were above average in economics and law, below average in German, and average in mathematics.

**Table 5.** Means and standard deviations of student competencies, school grades, and socioeconomic background by study program.

	EC	HS	Law	Med	ENT	OTHER	χ²(5)	ω
Economic knowledge and skills	0.56 (1.14)	−0.25 (0.89)	−0.31 (0.94)	−0.11 (0.86)	0.19 (0.87)	−0.26 (0.84)	28.4**	0.29
Interest in economics	0.42 (0.87)	0.01 (0.97)	0.07 (0.87)	0.25 (0.90)	−0.01 (0.90)	−0.27 (1.03)	7.7	0.15
Intrinsic motivation in economics	0.18 (0.91)	−0.12 (0.99)	0.42 (0.85)	0.10 (1.10)	0.02 (0.87)	−0.07 (0.98)	3.2	0.10
Value-oriented disposition regarding economics	0.37 (0.76)	0.02 (0.98)	0.09 (0.89)	0.24 (1.01)	0.01 (0.92)	−0.30 (1.08)	6.3	0.14
Attitude toward economics	0.47 (0.90)	−0.16 (0.91)	0.16 (0.92)	0.31 (0.85)	−0.07 (0.90)	−0.32 (1.05)	13.2*	0.20
Mathematical achievement	−0.17 (1.03)	−0.30 (0.87)	−0.28 (0.67)	0.54 (1.21)	0.44 (1.05)	−0.15 (0.89)	15.6**	0.22
First language skills	−0.36 (1.07)	0.06 (1.32)	0.10 (0.83)	0.02 (0.96)	−0.08 (0.75)	0.11 (0.81)	4.9	0.12
Cognitive ability	0.19 (0.79)	−0.25 (1.02)	−0.36 (1.18)	0.63 (0.72)	0.19 (0.97)	−0.21 (1.04)	16.2**	0.22
School grade: Mathematics	−0.19 (0.85)	−0.15 (0.96)	−0.33 (1.08)	0.44 (0.98)	0.35 (1.00)	−0.01 (0.92)	18.3**	0.24
School grade: German	−0.16 (0.75)	0.39 (0.90)	0.01 (1.02)	−0.04 (1.06)	−0.26 (1.00)	−0.04 (0.99)	22.3**	0.27
School grade: Economics and law	0.12 (0.92)	0.03 (0.84)	0.09 (0.84)	0.24 (1.14)	−0.14 (1.08)	−0.20 (0.98)	6.2	0.14
HISEI	−0.03 (1.11)	0.09 (0.99)	0.15 (0.87)	0.30 (0.62)	−0.06 (0.89)	−0.11 (1.08)	4.3	0.11

*Notes: HS: Humanities and social sciences; EC: Economics; Med: Medicine;*

*ENT: Exact, natural, or technical sciences; HISEI: Highest international socioeconomic index of occupational status (of parents).*

Numbers in parentheses represent standard deviations, *p *<* 0.05, **p *<* *0.01.*

When considering all achievement variables simultaneously in one test (economic competencies, mathematics performance, first language, cognitive abilities, and school grades), there were marked differences among the six groups (χ²(20) = 102.8, *p* < 0.01, ω = 0.56). Table 5 also shows the results of the univariate Wald tests.

#### Economic competencies as domain-specific competencies

Regarding the univariate results, post hoc analyses using Benjamini‒Hochberg adjustments for the p values (Benjamini and Hochberg, 1995; Benjamini and Yekutieli, 2001) were calculated, revealing significant differences between students in economics and those in other fields. The results show that students in economics had significantly higher economic knowledge and skills at the end of secondary education than did students in the other fields (HS: *d* = 0.80, Law: *d* = 0.81, Med: *d* = 0.64, ENT: *d* = 0.37, Other: *d* = 0.84). They also showed significantly higher values in attitude toward economics than students in the exact, natural, and technical sciences and other subjects (ENT: *d* = 0.70, Other: *d* = 0.80) and greater interest in economics than students in other subjects (*d* = 0.72).

#### Economic competencies as domain-independent competencies

Considering the mean differences regarding the other study groups, small to moderate effect sizes exist, with several groups of students showing above-average values in economic competencies. First, law students have above-average values in intrinsic motivation and attitude toward economics. Second, medical students have above-average values in interest in economics, intrinsic motivation, value-oriented disposition, and attitude toward economics. Third, students of exact, natural, and technical sciences show above-average economic knowledge and skills values. Regarding these values, students in law, medicine, and exact, natural, and technical sciences do not significantly differ from students in economics. However, these above-average mean values are insignificant when considering the Benjamini-Hochberg adjustment compared to humanities, social sciences, and other subjects.

To summarize the group differences in economic competencies, there are strong and significant differences between students in economics and students in humanities and social sciences, as well as students of other subjects, but there are almost no differences between economics students and students of law, medicine, and the exact, natural, and technical sciences, especially in terms of the motivational-affective dimensions of economic competencies. Students of humanities and social sciences, as well as students in other subjects (e.g., sports or teacher training programs, etc.), show below-average values in almost all dimensions of economic competencies.

#### Further group differences

In addition to economic competencies, some other group differences can be identified and are summarized in the following section. Concerning mathematical achievement, medical students and those of exact, natural, and technical sciences show the highest values. These are significantly higher than those of economics students (MED: *d* = 0.65, ENT: *d* = 0.59). Regarding verbal achievement, most study groups show average values, except for economics students. However, these differences are not significant when considering the Benjamini-Hochberg adjustment. Students of medicine show by far the highest values in cognitive abilities. Similar to mathematics and verbal achievement, there are also differences in school grades. For example, students of humanities and social sciences have the best grades in German, while students in medicine and exact, natural, and technical sciences have the best grades in mathematics. These school grades are significantly better than those of economics students. Considering school grades in economics and law, students of economics, law, or medicine have the best grades, while students of exact, natural, and technical sciences and students of other subjects have below-average grades. Finally, most students have an average socioeconomic background, while students of medicine or law score more highly.

### Prediction of study choices

In the following section, the previous section's bivariate results are further investigated using multinomial logistic regressions. To better understand the effects of economic competencies on university subject choices, four regression models were calculated (see Table 6), with each model building on the previous model (hierarchical regression). The first model shows the effects of economic competencies without any control variables. In the second model, the effect of economic competencies was controlled for background variables formulated in the EVT model, namely, gender, socioeconomic background, and the student's school profile (other vs. economics and law). To consider students’ academic abilities, the third model controlled for mathematical achievement, verbal achievement, and cognitive skills. Finally, these effects were further controlled for students’ school performance, as represented by standardized school grades. Odds ratios for each predictor were estimated as indicators of the probability of choosing a particular study program compared to choosing economics. Values above 1.00 indicate an increased likelihood of selecting a study program other than economics if an individual reaches higher values on the predictor. Values below 1.00 indicate the opposite. Since each model is a nonlinear model with a different number of variables, coefficients are not directly comparable across the four models (see, e.g., Mood, 2010).

**Table 6.** Prediction of major choices by economic competencies (multinomial logistic regression, as odds ratios).

	**Model 1**					**Model 2**					**Model 3**					**Model 4**				
	HS	Law	Med	ENT	OT	HS	Law	Med	ENT	OT	HS	Law	Med	ENT	OT	HS	Law	Med	ENT	OT
*Economic Competencies*																				
Economic knowledge and skills	**0**.54**	**0**.55*	**0**.45**	0.83	**0**.63*	**0**.**68^†^**	**0**.62*	0.68	0.96	0.77	**0**.68*	**0**.64*	**0**.**65^†^**	0.99	**0**.**76^†^**	**0**.60**	**0**.61**	**0**.63*	1.01	**0**.72*
Interest in economics	0.80	**0**.**46^†^**	0.76	0.56	**0**.**56^†^**	0.80	**0**.**48^†^**	0.74	0.54	**0**.**60^†^**	0.84	**0**.**55^†^**	0.83	0.63	**0**.**65^†^**	0.81	**0**.**58^†^**	0.83	0.65	**0**.**64^†^**
Intrinsic motivation regarding economics	1.24	**2**.61*	1.15	2.07	**1**.94*	1.11	**2**.33*	1.07	1.87	**1**.62*	1.11	**2**.07*	0.98	1.57	**1**.**53^†^**	1.20	**1**.91*	0.99	1.63	**1**.**60^†^**
Attitude toward economics	0.64	0.78	1.17	0.49	0.61	0.82	0.91	1.30	0.58	0.80	0.87	0.91	1.27	0.65	0.83	0.89	0.96	1.2 2	0.68	0.85
Value-oriented disposition (in economics)	1.02	1.08	1.00	0.98	0.85	0.80	1.00	0.98	1.01	0.82	0.92	0.97	0.98	0.98	0.82	0.86	0.89	0.98	0.97	0.79
*Background Variables*																				
Gender (0 = female, 1 = male)						**0**.64*	0.71	0.80	0.94	**0**.65**	0.76	0.83	0.79	0.90	**0**.74*	0.83	0.88	0.81	0.96	**0**.75*
HISEI						1.02	1.06	1.35	0.99	0.92	1.01	1.03	1.33	1.03	0.93	1.02	1.03	1.38	1.05	0.94
Advanced course (0 = other, 1 = economics and law)						0.87	1.09	**0**.61**	**0**.68*	**0**.80*	0.87	1.06	**0**.**75^†^**	**0**.**76^†^**	**0**.82*	0.90	1.09	**0**.73*	**0**.**76^†^**	**0**.84*
*Academic Abilities*																				
Mathematical achievement											0.98	0.99	1.44	**1**.75*	1.08	0.91	1.00	1.18	**1**.**50^†^**	1.02
German language skills											1.54	**1**.**54^†^**	1.22	1.19	**1**.46*	1.25	1.42	1.08	1.02	**1**.**35^†^**
Cognitive ability											0.76	0.75	1.41	0.79	**0**.**76^†^**	0.82	**0**.**71^†^**	1.41	0.75	0.78
*School Grades*																				
Mathematics																0.98	0.84	1.32	**1**.53*	1.13
German																**1**.46*	1.17	0.96	0.98	1.09
Economics and Law																1.09	1.13	1.10	0.83	0.94
*** AIC***	***1077***					***1071***					***1043***					***960***				

Notes: Reference group is economics; HS = humanities and social sciences; Med = medicine; ENT = exact, natural, or technical; OT = other;

Significant effects are highlighted in bold: **^†^** *p* < 0.10, *p < 0.05, **p < 0.01.

#### Economic competencies as domain-specific competencies

In Models 1 to 4, the strongest effects were found for advanced courses, economic knowledge and skills, interest, and intrinsic motivation regarding economics. Students with advanced courses in economics and law and students with higher economic knowledge and skills and interest in economics were more likely to study economics, holding other predictors constant. Considering the model with all control variables (see Model 4), there are still strong effects of economic knowledge and skills and advanced school courses. Based on these results, H1 was accepted regarding economic knowledge and skills but was rejected regarding motivational-affective dimensions of economic competencies. Furthermore, H4 was accepted.

Considering further variables, there are two main findings: (1) Students who had better school grades in German were more likely to study humanities and social sciences than economics, and (2) students with higher mathematics achievement and better school grades in mathematics were more likely to study exact, natural, and technical sciences than economics. Both results are in line with previous research (e.g., Guo et al., 2017; Parker et al., 2012; M.-T. Wang et al., 2013). Regarding these results, both H2 and H3 were accepted

#### Economic competencies as domain-independent competencies

In addition to the domain-specific effects of economic competencies described above, the domain-independent effects of economic competencies found within the (M)ANOVAs also become visible within the multinomial logistic regression models. First, students with higher intrinsic motivation are more likely to choose a study program other than economics. In this respect, students with higher intrinsic motivation are more likely to choose law. It is conspicuous that students with higher intrinsic motivation are also more likely to choose other subjects, which was not apparent within the bivariate analyses. In this regard, importantly, these are partial correlations for which correlations between independent variables are controlled. Similar to the bivariate analyses, the effects of economic competencies do not apply to all study programs in the same way. Considering Model 4, attitude toward economics and value-oriented disposition become insignificant. Furthermore, students with higher interest and intrinsic motivation in economics are not less likely to choose humanities, social sciences, medicine, or exact, natural and technical sciences.

## Discussion

In view of the growing importance of economic education for modern societies, the shift of economics education from purely domain-specific and vocational to an essential part of general school education and its role in twenty-first-century skills, researchers, politicians, and practitioners have given increasing attention to the importance of economic education at secondary and tertiary levels. Against this background, our paper has pursued two research objectives. The first is the justification of economic competencies as part of twenty-first-century skills and its expression of economic competencies among different study groups. The second is the domain-specific effects of economic competencies on the chosen study program. Based on a representative sample of Swiss students with two distinct time measurement points, this paper provides unique findings on economic competencies at the end of upper secondary schooling and toward higher education. Multivariate analyses of variances and hierarchical multinomial logistic regression models were used to calculate robust effects. The results are now summarized based on the two research questions of this paper.

### Research Question 1: How do the economic competencies as part of twenty-first-century skills differ between students in different study programs?

Based on a large body of research, we established economic competencies as an essential part of the skills individuals in modern societies should possess. At a societal level, the core argument is that economic challenges such as increasing globalization, economic dependencies, and economic crises require well-informed and responsible citizens who can understand and are willing to address these challenges. On an individual level, economic competencies have, in addition to better professional opportunities, a positive influence on the responsible handling of the private financial budget, which is increasingly necessary in response to privatization (e.g., Aprea et al. 2016). Here, we conclude that economic competencies are essential for future skills that must be acquired in modern society.

Considering the expression of economic competencies across the different study groups, bivariate analyses showed that the higher the economic competencies at the end of upper secondary school, the less likely learners are to study humanities, social sciences, or other subjects (except those with a higher intrinsic motivation). The effects are significantly smaller for the choice of law, medicine, and exact, natural, and technical sciences. Accordingly, it can also be assumed that learners with a positive attitude, a positive interest in, and a high motivation toward economics will be found more frequently in these study programs. This indicates that economic competencies are also comparatively strong for students of law, medicine, and the exact, natural, and technical sciences. In contrast, students of the humanities and social sciences, as well as those of other subjects, are deficient in these competencies. This begs the question of whether more specific interdisciplinary courses that address economic problems should be offered and evaluated specifically in these study programs.

### Research Question 2: How do students’ economic competencies at the end of upper secondary school predict their choice of an economics study program?

Consistent with EVT, students who had higher economic knowledge and skills (and therefore probably higher expectations of success) and were more interested in economics (higher intrinsic value) were more likely to choose economics instead of other fields of study after school. However, there were no significant effects of attitude toward economics or value-oriented dispositions and adverse effects of intrinsic motivation. Regarding attitude toward economics, it can be assumed that it primarily affects aspirations and only indirectly affects decisions (see, e.g., Ajzen, 1991; Fishbein and Ajzen, 1975), which cannot be further investigated. For intrinsic motivation, it can be assumed that high correlations with interest confound these effects. However, based on variance inflation factors (VIFs)^6^, assumptions regarding multicollinearity are not violated. The VIF values ranged from 1.2 (for advanced courses) to 3.2 (for interest in economics) and are, therefore, within an acceptable range (smaller 10.0).

The results make clear that economic competencies explain, to a significant extent, the prediction of the choice of an economics degree program. However, the domain-specific effects of economic competencies are almost exclusively valid for economic knowledge and skills. It is particularly interesting that the motivational-affective factors show no or only minor effects within the multivariate models. This is in contrast to previous findings regarding, for example, interest in mathematics and predicting STEM (e.g., Wang and Degol, 2013). Thus, the prediction of subject choice also shows that certain fields of study are chosen with equal frequency as economics when interest in economics and attitudes toward economics are more positive.

### Further findings

To control for the validity of the analyses and better understand the effects of economics competencies, we also controlled for further basal competencies and school grades, which are typically used to explain subject choice. Students with higher mathematical or verbal achievement and better school grades in mathematics or the first language are more likely to choose a study program other than economics (hypotheses H2 and H3). This strongly supports the assumption that economic competencies are independent of other domain-specific competencies or school-related experiences in other domains. In addition to economic competencies, the results showed that students who chose an advanced course in economics and law were more likely to study economics (see hypothesis H4). This indicates that students’ decisions about a study program might already be made at the lower and upper secondary school threshold when choosing an advanced school course. This is discussed in more detail below. Comparatively low gender effects showed that male students were only slightly more likely to select economics than female students, which is consistent with federal statistical data (OECD, 2017; SCCRE, 2023). These effects align with previous research and support the validity of the analyses in this article.

### Practical implications

Examining the global discussion about the relevance of twenty-first-century skills, at least two main issues can be identified from an educational perspective: (1) The development and adaption of national curricula to enable an alignment between twenty-first-century skills and curricular policies (see, e.g., Drake and Reid, 2018, 2020; Voogt and Roblin, 2012) and (2) the development of teacher education and teacher training to prepare teachers to meet the requirements of what has been dubbed the Fourth Industrial Revolution (Education 4.0; see, e.g., González-Pérez and Ramírez-Montoya, 2022; Urbani et al., 2017; for a review see also Teo et al., 2021). Considering current developments regarding these two issues, it remains unclear how to measure twenty-first-century skills and their determinants (van Laar et al., 2020a; Voogt and Roblin, 2012)^7^. This is also because most (or all) twenty-first-century skills, such as problem-solving, critical thinking, communication, etc., are domain-independent and are not explicitly taught (e.g., Tight, 2021; Voogt and Roblin, 2012). For higher education, Tight (2021) pointed out that many twenty-first-century skills, such as critical thinking, are already taught implicitly, although additional interventions to foster those skills are still recommended. Regarding teacher education, Teo et al. (2021: 15) identified at least two limitations: (1) understanding and developing frameworks to prepare teachers appropriately for the realities of the Industrial Revolution 4.0, and (2) the effective use of new technologies to prepare teachers. Against this background, it is evident that there is no general solution to teach these skills in teacher training or implement them into a national curriculum. This is because twenty-first-century skills consist of a complex conglomerate of skills that are domain-independent and strongly interdependent (see, e.g., Urbani et al., 2017).

Breaking down twenty-first-century skills into subordinate skills, such as economics as part of socio-cultural skills and citizenship (e.g., Voogt and Roblin, 2012: 309), might be an appropriate solution to understand better the different skills necessary to enable an alignment between twenty-first-century skills and national curricula as well as teacher training programs.

Based on our analyses of existing literature regarding the two issues mentioned above, no study has been conducted that explicitly analyzes economic competencies^8^ as part of twenty-first-century skills. Thus, to our knowledge, this study is the first to emphasize economic competencies as a vital aspect of twenty-first-century skills. Considering teacher education, studies point to significant deficits in the qualifications of economics teachers (for an overview, see, e.g., Compen et al., 2019). In addition, studies have also shown that an economics teacher's professional knowledge is a valuable predictor of student economic competencies (for an overview, see, e.g., Jüttler and Schumann, 2022b). Indeed, this paper underlines the need for further research to observe not only students’ economic competencies but also those of upper-secondary and tertiary levels teachers in more detail. Since the present study did not explicitly considered these educational aspects, suggestions for future research are discussed below.

### Limitations

In addition to our findings and their implications, some limitations must be acknowledged. First, from a methodological perspective, it must be remembered that the data were collected between 2011 and 2016. Since that time, various structural changes have occurred, especially at school level. A central change in this context is, for example, a recent announcement by the Swiss Conference of Cantonal Ministers of Education stating that “Economics and Law” will become a core subject at GS (see EDK, 2023). As a result, all future GS students can be expected to address economic problems in their learning career. Consequently, findings of this study can probably only be compared with future cohorts to a limited extent. However, this change also underlines our claim that economic content is not only domain-specific but also influences the general study ability of students (ibid.). Another significant development in recent years is the declining number of students, which has brought greater competition between the various universities and a redistribution of student quotas between the different subject groups (e.g., SCCRE, 2023). This paper cannot predict how these developments might influence the effects of economic competencies on a student's choice of a study program. In addition, the contexts and issues used in the test to measure economics knowledge and skills used in this paper are also outdated and must be renewed in light of emerging economic issues.

Second, the results of this paper are restricted by the measurement of economic competencies. In this regard, the additional facets of economic competencies refer to the school subject of economics and law and not to the domain itself (e.g., “During lessons in economics and law, time often flies by,” rather than “when learning about economics or business administration, time often flies by.”).

Third, self-concept was not considered in addition to student achievement in the different domains. Although domain-specific achievement correlates with the corresponding self-concept (e.g., Marsh et al., 2005), student perceptions of their abilities are more proximal predictors of course selection (Eccles and Wigfield, 2002).

Fourth, as previously mentioned, study courses were categorized into relatively large groups because of the sample size. For instance, economic study courses were not separated from business administration courses, meaning some effects may have been biased or underestimated. This could also explain the intrinsic motivation findings, as some intersections exist between economics and law, for example, business and commercial law. Similarly, it is difficult to interpret the results for the group of students in the “other” field because of its considerable heterogeneity.

Fifth, our analysis does not consider the point at which the first course was chosen. As some students went abroad or completed an internship after school, their domain-specific interests and perceptions may well have changed. Thus, our analyses cannot control for the exact ages of transitions.

Sixth, although student drop-outs could be appropriately addressed by inverse-probability weighting (see Dropout at T2 and weighting), we could only use complete information of T1 variables to calculate appropriate weightings. This is a common and well-developed method to address unit non-response. However, we cannot fully guarantee that these represent accurate distributions since the calculation of the weightings is reduced to the T1 variables that could have been identified as drop-out variables.

Finally, considering the discussion about twenty-first-century skills, it is difficult to determine which students in certain groups have received more education in economics and are, therefore, better prepared than other students to engage with future economic issues (e.g., by being more inclined to be informed about economic issues or seeing economic issues as more important). Furthermore, there are many more dimensions of twenty-first-century skills that we did not address (e.g., critical thinking). With this in mind, our study does not and cannot claim to explain how students’ twenty-first-century skills differ among those in different study groups, a matter discussed in more detail in the following section.

### Suggestions for future research

Some research desiderata can be derived from our analyses. First, economic competencies are just one small part of an extensive catalogue of twenty-first-century skills formulated by researchers (see González-Pérez and Ramírez-Montoya, 2022). Against this background, it is unclear how these specific competencies interrelate with subordinate skills, such as critical thinking, problem-solving, ethical behavior, or communication. There might be positive interrelations between some of these subordinate skills and dimensions of economic competencies, for example, between problem-solving and economic skills or between ethical behavior and value-oriented dispositions. Thus, it would also be of great interest explicitly to test students for a larger set of twenty-first-century skills to control for the effects of economic competencies. However, as mentioned in the theoretical section, many current definitions of twenty-first-century skills remain unclear and cannot be measured (e.g., Daumüller and Wisniewski, 2023). For this reason, an approach in which a clear frame of reference within twenty-first-century skills (such as societal relevant economic issues) is given might be – both from a theoretical and a methodological perspective – the more appropriate one.

Second, it is unclear how different basic competencies, such as mathematics or the first language, interact with economic competencies regarding study choices. Although interaction terms did not significantly improve the regression model^9^, how economic competencies formed in interaction with other competencies during school (see, e.g., Marsh et al., 2015) remains unclear. Thus, for future research, it would be of great interest to examine twenty-first-century skills not only at the threshold between school and university but also during school to explain their origins and interactions with economic competencies over time. This aspect also becomes visible when considering the effects of the advanced course. Based on this, it seems that many students have already chosen a study program when they select an advanced course, which means that the selection process might occur at an earlier point in time (rather than on leaving upper secondary school). Accordingly, competencies, interests, aspirations, and other issues should be considered before choosing an advanced course.

Third, a primary question is how students gain economic competencies through school education. As such, observations of learning and instruction in business and economics are important tasks for future research.

Fourth, this paper cannot explain how to promote students’ economic competencies in higher education. Although differences between groups of students may lead one to assume differences in the corresponding level of general economic preparation for future economic issues, to tackle deficits systematically, it is necessary to develop specific interdisciplinary courses that students can select or have to take during their studies. Several classes that train core twenty-first-century skills (e.g., presentation and communication skills) are often offered by universities (for “key skills,” see, e.g., Fallows and Steven, 2000). However, to our knowledge, these programs neither include nor address economic knowledge and skills or problem-solving tactics regarding economic issues.

Finally, as this paper has focused on the justification of economic competencies as part of twenty-first-century skills and the meaning of economic competencies in the transition from general upper-secondary to tertiary level, it is not possible to derive explicit adjustment recommendations, such as changes to curricular planning or teacher training. As a result, this study can only provide some initial findings and point to the necessity of further research in which (school) curricula and teacher training programs are explicitly considered.

### Conclusion

Despite some unanswered questions, this paper presents new findings about the long-term effects of economic competencies at the end of upper secondary school concerning the choice of further study programs. It draws on a broad understanding of economic competencies with multidimensional cognitive and motivational-affective dimensions. Economic competencies are understood as societally relevant knowledge based on a test that uses newspaper articles to ascertain how well citizens in modern society can participate responsibly in economic issues and form sound opinions. This is different from most other studies, which only focus on basic knowledge in economics or curricular tests without any link to (current) societal issues (see the underlying definition of economic competencies in the theoretical section and the measurement of economic competencies in the description of the instruments of this paper).

Our study answers research questions of high social and educational relevance, its most important contribution being that it underlines the importance of general economic education beyond simple improvements to personal finance arrangements or the understanding of basic economic principles. General economic education supports the self-selection process of deciding to study economics and is, therefore, domain-specific. In contrast, this paper supports the assumption that economic competencies are essential to twenty-first-century skills. Against this background, teaching and reflecting societally relevant economic content should be a core aim of vocational and general education at secondary and tertiary levels. The goal is not only to educate students to become economically competent citizens but also to provide them with basic skills they can use in private, professionally, and when engaging with society.

## Notes

1. Related study courses, e.g., business administration, are subsumed within economics in this paper.

2. It is important to note that the terms economic competencies and economic education are often used interchangeably. In this study, we are referring specifically to the concept of economic literacy that is closest to the concept of economic education. For a discussion of these different concepts and their relationships, see, e.g., Cortina (2016).

3. Initial findings can be found in Jüttler and Schumann (2019, 2022a, 2022b).

4. Advanced courses are Economics and Law, Ancient Languages, Modern Languages, Physics & Mathematics, Biology & Chemistry, Philosophy/Pedagogy/Psychology, Art and Music.

5. See https://www.soziologie.uni-konstanz.de/hinz/surveylab/.

6. To calculate the VIF values, it was necessary to translate the multinomial logistic regression into a comparable linear model. Therefore, we implemented study choice as a dichotomous dependent variable (0 = other study program, 1 = economics) within a linear regression model. Consequently, the VIF values are only approximate calculations. However, since the values are far from 10, they can still be considered valid.

7. Although the measurement is often criticized, there are different approaches to measuring 21^st^-century skills, e.g., digital skills (see, e.g., van Laar et al., 2020b; Rubach and Lazarides, 2021).

8. Exceptions can be found in studies dealing with financial literacy.

9. Additional models considered interaction effects among economic knowledge and skills, mathematical achievement, first language, and cognitive ability. However, these models did not lead to significant improvements in terms of model fit.

## Appendix

**Table A1.** Original sample (weighted).

	Classes	Students	Gender		Age	
			Female	Male	M	SD
GS (Economics and Law)	42	193	81 (42%)	112 (58%)	18.7	0.9
GS (Other)	37	1222	768 (63%)	454 (37%)	18.6	0.9
Total	79	1415	849 (60%)	566 (40%)	18.6	0.9

*Notes: GS* *=* *Baccalaureate (grammar) school; M* *=* *mean; SD* *=* *standard deviation.*

**Table A2.** Means and standard deviations of students at T1, T2, and dropout students.

	Unweighted: Sample T1*	Unweighted: Dropout**	Unweighted: Sample T2**	Weighted: Sample T2**
Economic knowledge and skills	0.00 (1.00)	−0.10 (0.99)	0.36 (0.95)	0.26 (0.94)
Interest in economics	0.00 (1.00)	−0.05 (1.00)	0.18 (0.97)	0.16 (0.99)
Intrinsic motivation in economics	0.00 (1.00)	−0.04 (0.99)	0.15 (1.02)	0.10 (1.07)
Attitude towards economics	0.00 (1.00)	−0.06 (0.99)	0.21 (1.01)	0.16 (0.99)
Value-oriented disposition regarding economics	0.00 (1.00)	−0.05 (1.00)	0.16 (0.98)	0.12 (0.96)
Mathematic performance	0.00 (1.00)	−0.11 (0.99)	0.35 (0.93)	0.00 (1.02)
First language skills	0.00 (1.00)	−0.09 (0.99)	0.28 (1.00)	−0.08 (1.18)
Cognitive abilities	0.00 (1.00)	−0.09 (1.02)	0.27 (0.90)	0.00 (0.92)
School grade: Mathematics	0.00 (1.00)	−0.04 (0.99)	0.13 (1.01)	0.08 (0.97)
School grade: German	0.00 (1.00)	−0.04 (1.01)	0.13 (0.95)	0.08 (0.92)
School grade: Economics	0.00 (1.00)	−0.05 (1.01)	0.17 (0.95)	0.12 (0.93)

*Notes: Standard deviations are shown in parentheses*.

**All values are z-standardized. **All values are standardized based on the distribution at T1.*

*The values of students at T1 are z-standardized (M* *=* *0.0, SD* *=* *1.0).*

### A1. Exemplary Item #1 to Measure Economic Knowledge and Skills *(translated)*

What is meant by the term “reindustrialization” in Switzerland?

- The turnover of Swiss industrial companies increases compared to the previous period.
- The production of industrial goods is increasing in Switzerland.
- Swiss production capacity is better utilized compared to the previous period.
- Swiss production capacity of industrial goods manufacturing is increasing.

### A2. Exemplary Item #2 to Measure Economic Knowledge and Skills *(translated)*

Which elements mentioned in the article are more likely to be assigned to a state-directed economy, and which to a free market economy? Name one element each and briefly justify your classification.

**Table:**

State-directed economy (including justification)	Free market economy (including justification)
	
